# Upstream Stimulating Factors 1 and 2 Enhance Transcription from the Placenta-Specific Promoter 1.1 of the Bovine *Cyp19 *Gene

**DOI:** 10.1186/1471-2199-11-5

**Published:** 2010-01-18

**Authors:** Rainer Fürbass, Wolfgang Tomek, Jens Vanselow

**Affiliations:** 1 Research Unit Molecular Biology, Research Institute for the Biology of Farm Animals (FBN), Wilhelm-Stahl-Allee 2, 18196 Dummerstorf, Germany; 2 Research Unit Reproductive Biology, Research Institute for the Biology of Farm Animals (FBN), Wilhelm-Stahl-Allee 2, 18196 Dummerstorf, Germany

## Abstract

**Background:**

Placenta-derived oestrogens have an impact on the growth and differentiation of the trophoblast, and are involved in processes initiating and facilitating birth. The enzyme that converts androgens into oestrogens, aromatase cytochrome P450 (P450arom), is encoded by the *Cyp19 *gene. In the placenta of the cow, expression of *Cyp19 *relies on promoter 1.1 (P1.1). Our recent studies of P1.1 *in vitro *and in a human trophoblast cell line (Jeg3) revealed that interactions of placental nuclear protein(s) with the E-box element at position -340 are required for full promoter activity. The aim of this work was to identify and characterise the placental E-box (-340)-binding protein(s) (E-BP) as a step towards understanding how the expression of *Cyp19 *is regulated in the bovine placenta.

**Results:**

The significance of the E-box was confirmed in cultured primary bovine trophoblasts. We enriched the E-BP from placental nuclear extracts using DNA-affinity Dynabeads and showed by Western blot analysis and supershift EMSA experiments that the E-BP is composed of the transcription factors upstream stimulating factor (USF) 1 and USF2. Depletion of the USFs by RNAi and expression of a dominant-negative USF mutant, were both associated with a significant decrease in P1.1-dependent reporter gene expression. Furthermore, scatter plot analysis of P1.1 activity *vs. *USF binding to the E-box revealed a strong positive correlation between the two parameters.

**Conclusion:**

From these results we conclude that USF1 and USF2 are activators of the bovine placenta-specific promoter P1.1 and thus act in the opposite mode as in the case of the non-orthologous human placenta-specific promoter.

## Background

**Figure 1:**
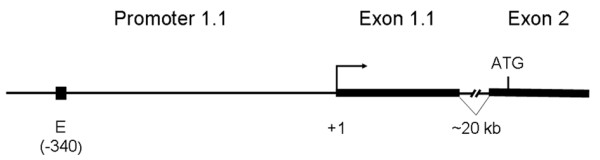
**Schematic representation of the bovine placenta-specific promoter P1.1**. The promoter is shown as black horizontal line with the E-box (-340) shown as a black box. The transcription start site is marked by +1 and an arrow. Exons 1.1 and 2 are drawn as thick black lines, the ~20 kb intron between them is indicated. The translation start site within exon 2 is marked by the ATG codon.

Ruminant species produce oestrogens in the cotyledons of their placentas. The functions of placental oestrogens change during gestation. First, together with progesterone, they are local activators of trophoblast growth and differentiation, acting via intracrine, autocrine or paracrine mechanisms (reviewed by [1]). Second, an oestrogen peak near term triggers endocrine and paracrine processes involved in the initiation of parturition, cervical softening and dilatation, and increasing myometrial contractibility (reviewed by [2]). In ruminants, low oestrogen levels and a reduced prepartal oestrogen peak are associated with an increased abortion rate, dystocia, and placental retention [3-5]. Oestrogens are synthesised by aromatisation of androgen precursors, which is catalysed by the enzyme aromatase cytochrome P450 (P450arom; EC 1.14.14.1). Oestrogen biosynthesis in various tissues depends on the expression of the P450arom-encoding *Cyp19 *gene, which involves tissue-specific promoters. Tissue-specific *Cyp19 *transcripts contain at their 5'ends unique untranslated exons, which, during processing, are spliced to a common acceptor site just upstream of the coding region [6]. Accordingly, these tissues express identical P450arom proteins. The placenta-specific *Cyp19 *promoter in the cow is P1.1 (Figure 1). Interestingly, placenta-specific promoters in humans [7] and even in sheep [8] are not orthologous to P1.1.

To elucidate the regulatory mechanisms behind the expression of *Cyp19 *in the bovine placenta we have analysed P1.1 *in vitro *and by reporter gene experiments. We found that the E-box element at position -340 was required for full promoter activity in Jeg3 cells, which served as a model for trophoblast cells. Moreover, it has been demonstrated by electrophoretic mobility shift assays (EMSA) that a placental transcription factor could specifically bind to the E-box (-340) [9]. The human placenta-specific promoter has also been extensively studied by others. It has been demonstrated in transgenic mice that a sequence of only 500 bp in this promoter could mediate placenta-specific expression of a transgene [10,11]. In cultured human trophoblast cells under hypoxic conditions, *Cyp19 *expression was inhibited by the hypoxia-inducible transcription factor MASH-2, though its binding to the *Cyp19 *promoter was not demonstrated [12]. The inhibitory effects of hypoxia and MASH-2 on *Cyp19 *expression in the human placenta turned out to be mediated by binding of the transcription factors USF1 and USF2 to two E-box elements [13,14]. Furthermore, surveys of the DNA methylation status and of the chromatin structure of bovine and ovine placenta-specific promoters provided evidence of the involvement of epigenetic mechanisms in tissue-specific and developmental regulation of *Cyp19 *expression [15-17].

The aim of the present work was the identification and characterisation of the placental transcription factor(s) binding to the E-box (-340) element (E-BP) and to fully define these interactions as a step towards understanding the regulatory mechanism underlying the expression of *Cyp19 *in the bovine placenta. To this end, we developed a procedure to enrich the E-BP from placental nuclear protein extracts by using immobilised E-box target DNA. Western blot analysis and supershift experiments revealed that the E-BP consists of the transcription factors USF1 and USF2. Their functional significance with regard to P1.1-dependent gene expression was demonstrated by RNAi-mediated knockdown experiments and by the expression of a dominant-negative USF mutant.

## Results

### P1.1 activity in primary bovine trophoblast cells

**Figure 2:**
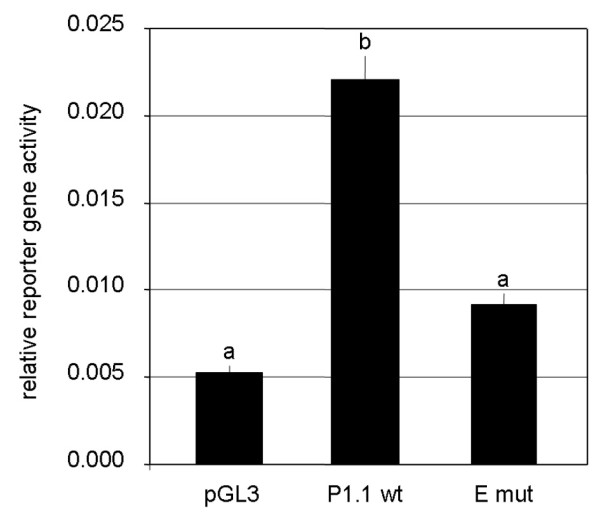
**E-box (-340) is essential for transcriptional activity of P1.1 in primary bovine trophoblast cells**. Cells were transiently transfected with a promoterless luciferase reporter gene plasmid (pGL3), which served as a control, or with luciferase reporter gene plasmids containing the proximal sequence of P1.1 and part of the untranslated exon 1 (base pairs -404 to +113) with either a wild type (P1.1wt) or a mutated E-box (-340) motif (Emut), along with plasmid CMV-*lacZ*. Activities of the luciferase and *lacZ *reporter genes were measured 24 h after transfection. The quotient of the luciferase and β-galactosidase activities was calculated to normalise for transfection efficiency. Results are expressed as the mean ± S.E.M of n = 3 experiments. Different letters above the columns indicate significant differences (p < 0.05).

The transcriptional activity of P1.1 was reassessed in a recently established tissue culture model of primary bovine trophoblast cells (pbTC) [18]. Trophoblast cells were transiently transfected with P1.1 reporter gene plasmids containing either a wild type or a mutated E-box motif, as described in Materials and Methods. Cells were harvested 24 h later, and reporter gene activities were measured. The wild type P1.1 clearly enhanced reporter gene expression in pbTC (Figure 2). Upon inactivation of the E-box (-340) by point mutations the reporter gene activity dropped significantly, and reached a level not significantly different from that of the promoterless control plasmid. The results show that the E-box (-340) element is required for full promoter activity in bovine trophoblasts.

### The E-box binding protein is composed of the transcription factors USF1 and USF2

**Figure 3:**
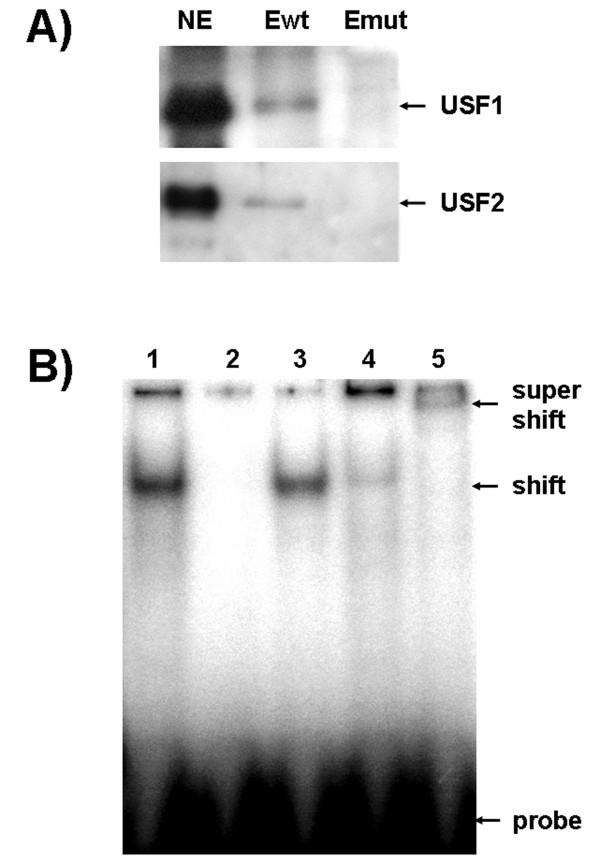
**The E-box binding factor is composed of USF1 and USF2**. **A) **Western blot analysis of proteins eluted from immobilised target DNA containing a wild type (Ewt), or a mutated E-box motif (Emut). Protein samples were subjected to SDS-PAGE alongside the nuclear extract (NE). Gels were blotted and incubated with commercial anti USF1- and anti USF2-IgG. Both antibodies interacted with nuclear extracts and eluates from the wild type, but not from the mutated target oligonucleotides. **B) **EMSA experiments with the labelled E-box probe and bovine placenta-derived nuclear extracts. With nuclear extract, a complex (shift) formed (lane 1). Specificity of the complex was demonstrated by competition with a 100-fold excess of the unlabelled probe (lane 2) or a mutated probe (lane 3). Addition of anti USF1 antibodies strongly reduced the complex (lane 4). In the presence of anti USF2 antibodies, a supershift complex formed (lane 5). There is no evidence for more than one specific complex, suggesting that the probe is bound by USF1/2 heterodimers.

A procedure was developed to enrich the E-BP from placental nuclear protein extracts. To optimise the yield of a specific E-BP/DNA complex, and to reduce "unspecific" protein binding to non-E-box sites of the probe, unspecific competitor DNA was added to the binding reactions. Furthermore, we took advantage of an observation made during pilot experiments, that the E-BP was resistant to heat treatment at 65°C for 10 min. While most of the original E-BP activity was preserved, unspecific complexes were no longer detectable during EMSA experiments (not shown). In small-scale experiments, heat-treated extracts were incubated with E-box target DNA, which had been immobilised to paramagnetic Dynabeads. As a control, experiments with mutated target sites were performed in parallel. The eluted proteins were analysed by SDS-PAGE. A 35-kDa protein band was consistently detected with proteins eluted from the wild type, but not from the mutated E-box target sites (not shown). To identify the protein(s), the 35-kDa band was cut out of the gel and processed for MALDI-TOF analysis. The obtained spectra, however, were not informative, due to contamination of the samples by BSA, which was an indispensable component of the elution buffer. Omitting BSA resulted in the loss of the 35-kDa band (not shown). Nevertheless, the described features of the E-BP strikingly resembled those of the transcription factors USF 1 and USF2 [19-21]: First, E-BP and USF proteins bind to a CACATG core E-box element; second, USF proteins are the only known heat-stable E-box-binding transcription factors; and third, the molecular weights of bovine USF proteins as deduced from their cDNA sequences (NP_001001161 and 001001162, respectively) range from 33.4 to 36.9 kDa. We therefore hypothesised that the E-BP might be USF1 and/or USF2. To demonstrate this experimentally, the eluted proteins were subjected to Western blot analysis using commercial anti USF1- and anti USF2-IgG. As shown in the Figure 3A, both antibodies visualised a 35-kDa band eluted from the wild type E-box targets. In contrast, no signal was obtained with eluates from mutated E-box targets. In supershift EMSA experiments both antibodies, but not an unrelated IgG (not shown), interacted with the E-BP complex. There was no evidence for more than one single complex suggesting that the E-BP is likely a heterodimer of USF1 and USF2 (Figure 3B). Taken together, our results demonstrate a specific interaction of the transcription factors USF1 and USF2 with the E-box (-340) motif of the placenta-specific promoter P1.1.

### Functional significance of USF1 and USF2 for P1.1 activity

**Figure 4:**
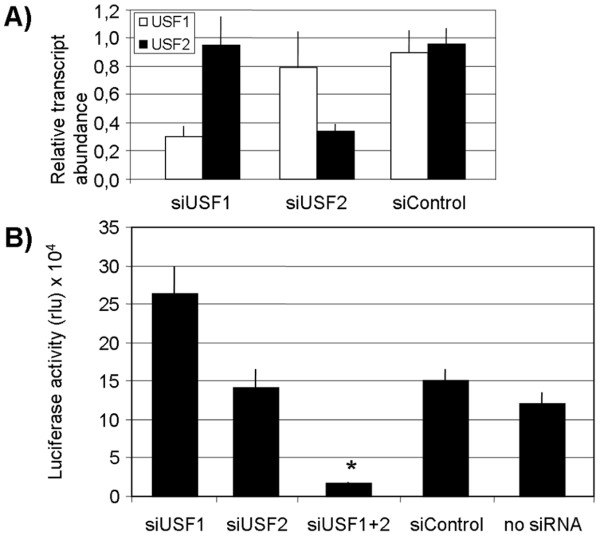
**siRNA-mediated double knockdown of USF1 and USF2 reduces P1.1 activity**. **A) **To examine the transcript specificity of knockdowns, Jeg3 trophoblast cells were transfected with pools of siRNAs targeting USF1 (siUSF1), USF2 (siUSF2) or a siControl pool, which does not target any known transcript. Mock-transfected cells treated only with the transfection vehicle were used to normalise measurements. USF transcripts were quantified by real-time PCR 24 h after transfection. The relative transcript abundance was calculated by dividing measurements from siRNA treated cells with measurements from mock-transfected cells. Mean values ± S.E.M. of n = 3 experiments are shown. **B) **To analyse the effect of USF knockdown on P1.1-dependent transcription, Jeg3 cells were transiently transfected with the P1.1-luciferase reporter gene plasmid along with siRNAs (siUSF1, siUSF2 or siUSF1+USF2). Jeg3 cells transfected with a non-targeting siRNA (siControl), or without siRNA served as controls. Luciferase activities were measured 72 h after transfection. Results (relative light units, rlu) are shown as means ± S.E.M. of at least n = 3 experiments. Only the double knockdown resulted in a significant reduction of the reporter gene activity (p < 0.05), as indicated by an asterisk.

We used an RNAi-based approach to assess the function of both USF transcription factors with regard to P1.1 activity. Pools of siRNA targeting *USF1 *or *USF2 *transcripts were transfected into Jeg3 trophoblast cells, and the transcripts were then measured by quantitative real-time PCR (qPCR). As shown in Figure 4A, both USF siRNA pools specifically knocked down their respective USF transcripts. A control siRNA pool, which does not target any known transcript, did not affect the USF transcripts. Then, we transiently cotransfected Jeg3 cells with the USF siRNA pools, either individually or simultaneously, and with a reporter gene plasmid bearing P1.1, to measure the effects of USF knockdowns on P1.1-dependent transcription. The results are summarised in Figure 4B. Interestingly, transfection of either USF1 or USF2 siRNA alone did not reduce P1.1 activity. Unexpectedly, USF1 knockdown even increased the promoter activity. However, the double knockdown of *USF1 *and *USF2 *transcripts was associated with a significant decrease in P1.1-dependent reporter gene expression. Hence, the two factors could substitute for each other, at least in the case of the reporter gene experiments in Jeg3 cells.

**Figure 5:**
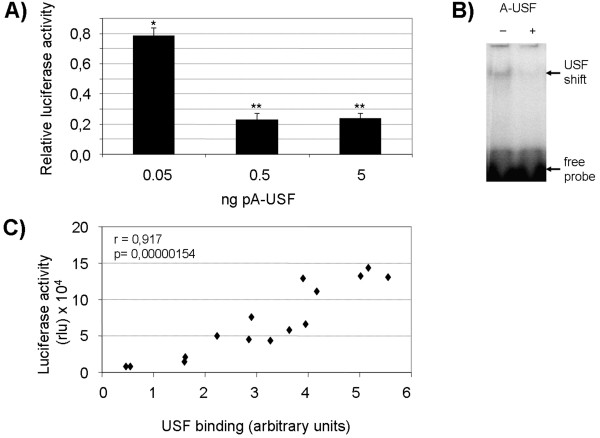
**P1.1-mediated gene expression is positively correlated with USF binding to the E-box (-340)**. **A) **Transient cotransfection assays in Jeg3 cells of the P1.1-luciferase reporter gene plasmid in the presence of the indicated amounts of the dominant-negative A-USF construct. The luciferase activities were measured 24 h after transfection. To normalise measurements, the results from analogous cotransfection experiments with the empty expression plasmid were arbitrarily set to 1. Mean values ± S.E.M. of n = 3 experiments are shown. Differences between A-USF transfected cells and cells transfected with the empty expression vector were significant, as indicated by asterisks (* p < 0.05; ** p < 0,001). **B) **representative EMSA experiments with the labelled E-box probe and Jeg3 cell extracts left from reporter gene analyses. Jeg3 cells were transiently transfected with the P1.1-luciferase reporter gene plasmid plus 5 ng of the empty expression vector (- A-USF) or 5 ng of the A-USF expression plasmid (+ A-USF). In the presence of A-USF, binding of USF to the E-box probe is impaired. **C) **Scatter plot of luciferase activity *vs. *USF binding. Data points represent reporter gene and USF binding activities of individual extracts from reporter gene experiments with different amounts of A-USF- or empty expression plasmids. USF binding was quantified by applying the Image Quant software to EMSA experiments similar to the one shown in panel B. Data were statistically analysed with the Pearson product moment test. The correlation coefficient, r, and the p value are indicated.

To analyse more directly if USF factors indeed bind to the E-box (-340), and thereby activate P1.1-dependent transcription, we cotransfected Jeg3 cells with a P1.1-luciferase reporter gene vector and an expression plasmid for a dominant-negative USF mutant, A-USF [22]. A-USF specifically dimerises with endogeneous USF1 and USF2 and efficiently prevents USF binding to DNA [22]. Indeed, upon introduction of various amounts of the A-USF construct into the Jeg3 cells, P1.1-driven luciferase reporter gene activity was reduced in a dose-dependent manner (Figure 5A). Lysates used for reporter gene measurements were also analysed by EMSA experiments, which revealed that A-USF indeed inhibited the formation of the USF/E-box binding complex (Figure 5B). To examine the relationship between P1.1 activity and USF binding to the E-box (-340), we plotted measurements of the reporter gene activity *vs. *USF/DNA complex intensity, as determined by EMSA experiments similar to the one shown in Figure 5B. Hence, in the scatter plot shown in Figure 5C, each data point represents measurements of both parameters in one given lysate. A strong positive correlation between the two parameters was observed.

## Discussion

### The E-box (-340) is required for P1.1-dependent transcription in pbTC

We studied the functional relevance of the E-box (-340). Results from reporter gene experiments with pbTC are in line with those of our earlier analyses with Jeg3 cells [9], but, unexpectedly, there were also remarkable differences. In pbTCs, the reporter gene activity significantly dropped upon inactivation of the E-box, reaching the level produced by the promoterless control plasmid. In contrast, our previous studies in Jeg3 cells had revealed that P1.1 activity dropped by only about 50%, and was still significantly higher than the reporter activity of the pGL3 control plasmid. Only additional mutations in the hexameric motif at position -268 completely abrogated P1.1 activity [9]. Hence, the hexameric motif (-268) is not needed for full P1.1 activity in pbTC. This conclusion is also supported by our observation in EMSA experiments that only nuclear extracts from Jeg3 cells, not from bovine placenta, yielded specific binding complexes with a probe containing the hexameric motif (not shown).

### The E-BP is composed of USF1 and USF2

Our results from supershift EMSA experiments and Western blot analysis of the proteins eluted from the immobilised target oligonucleotides show that the ubiquitously expressed bHLH-leucine-zipper transcription factors USF1 and USF2 bind to the E-box (-340), most likely as heterodimers. In most tissues and cell lines, USF1/USF2 heterodimers represent the major USF species [22]. The functional significance of USF binding to the E-box (-340) is emphasised by results of RNAi-mediated knockdown experiments. Interestingly, P1.1-dependent reporter gene activity was only decreased by double knockdown of the two USFs, suggesting at least partially overlapping functions of these two transcription factors. Similar observations have been reported by others studying effects of USF gene targeting in mice [21]. During single knockdown experiments, we did not observe effects with USF2 siRNA, but, surprisingly, reporter gene activity increased upon transfection of USF1 siRNA. The underlying mechanism, however, is not yet understood. The use of the dominant-negative A-USF protein gave a direct analysis of the effects of USF binding to the E-box (-340) on the transcriptional activity of P1.1. This analysis revealed a strong positive correlation between the two parameters. Taken together, different kinds of experimental challenges (e.g. the *trans*-acting depletion of USF1 and USF2 transcripts or proteins and the *cis*-acting E-box mutations), provoked consistent effects on P1.1 activity in the trophoblast cell culture model, emphasising the potential importance of USF/E-box-interactions for P1.1-dependent *Cyp19 *expression in the bovine placenta.

### How can ubiquitous USF transcription factors act in a cell/tissue-specific manner?

Although USF1 and USF2 are ubiquitously expressed transcription factors [23], P1.1-derived *Cyp19 *transcripts were predominantly found in the cotyledons of placentas [6]. However, both USF proteins are involved in cell/tissue-specific expression or developmental regulation of other genes as well, among them *Fshr *and *SF1 *[24,25]. But the mere presence of USF proteins is not sufficient for productive interactions with P1.1, because a second E-box motif at position -56, despite being an efficient binding site during EMSA experiments, proved to be irrelevant for promoter activity *in vivo *[9]. Hence, possible additional conditions, which could include appropriate spacing with regard to other binding motifs, presence of other transcription factors or of cofactors, and accessibility of binding sites, must be fulfilled to achieve functional interactions. A highly conserved domain shared by USF1 and USF2, termed the USF-specific region, putatively mediates cell-type-specific gene expression by interactions with a specialised coactivator [22]. Accordingly, USF-activated *Cyp19 *transcription in the placenta via P1.1 might well involve additional, yet unknown transcription factors or cofactors that are not present in other tissues. On the other hand, confinement of USF activities to a set of cell-type/tissue-specific or developmentally regulated promoters could be mediated by epigenetic factors, such as DNA methylation and chromatin structure. Our recent results mapping DNaseI hypersensitive sites within P1.1 support this assumption [15], and our finding in bovine placentomes that P1.1 DNA was unmethylated in the *Cyp19*-expressing cotyledons, but methylated in the non-expressing caruncles [17]. In sheep DNA methylation and chromatin accessibility of the placenta-specific *Cyp19 *promoter correlated with expression levels as well [16]. Interestingly, methylation of an E-box (CACGTG) prevented USF1/2 binding and abrogated *Fshr *transcription [26]. In the case of *Cyp19*, however, USF binding to P1.1 cannot be prevented by methylation of the E-box (-340) because its core sequence (CACATG) does not feature a potential CpG methylation site.

### USF binding to placenta-specific promoters from human and bovine *Cyp19 *has opposite effects

Several species, among them human, cattle and sheep, express *Cyp19 *in their placentas. They use different, non-orthologous placenta-specific promoters, which are located 100, 20 and 0.4 kb, respectively, upstream from the coding sequence [8,27,28]. Comparative analysis of their functional protein/DNA interactions might readily reveal transcription factors and respective promoter elements that are relevant for placenta-specific *Cyp19 *expression. Indeed, USF1 and USF2 interact with the bovine and human placenta-specific promoters, but with very different effects. The bovine E-box (-340) clearly activated the P1.1-dependent reporter gene expression in pbTC and also in Jeg3 cells [9]. In contrast, binding of USF to two E-box sites, at -58 of the human promoter and +26 in the adjacent untranslated exon, inhibited *Cyp19 *expression in human cytotrophoblasts [13]. Also in Jeg3 cells cultured in a normoxic environment, comparable to that used in our study, siRNA mediated double knockdown of USFs resulted in markedly increased levels of *Cyp19 *mRNA [14]. Hence, the observed effects of USF binding depend on the context of the promoter.

## Conclusions

In this study we show that i) the E-box (-340) element of the placenta-specific *Cyp19 *promoter P1.1 is required for full promoter activity in bovine trophoblasts and that ii) the transcription factors USF1 and USF2 interact with the E-box (-340) and thereby activate P1.1-dependent transcription. This suggests that USF1 and USF2 act in the opposite way on the bovine P1.1 and the (non-orthologous) human placenta-specific promoter. The results provide new insights into the molecular mechanisms that are involved to regulate P1.1-dependent transcription in bovine trophoblasts.

## Methods

### Bovine trophoblast cell culture, transient DNA transfection and reporter gene analysis

Cultures of primary bovine trophoblast cells (pbTC) were established as described in [18]. The P1.1 reporter gene plasmid, which is a derivative of the pGL3 basic vector (Promega, Mannheim, Germany) carrying the P1.1 sequence from positions -404 to +113, and plasmid CMV-*lacZ *are described elsewhere [9]. For DNA transfection a mix of DNA (250 ng reporter gene plasmid and 5 ng CMV-*lacZ *vector), Lipofectamine Transfection Reagent, and Plus Reagent (Invitrogen, Karlsruhe, Germany) were added to a nearly confluent layer of pbTC in 24-well plates. Activities of the luciferase and *lacZ *reporter genes were measured 24 h after transfection in a luminometer instrument (Lumat LB9501, Berthold, Wildbad, Germany) using the Dual Light System (Applied Biosystems, Darmstadt, Germany) according to the supplier's instructions. To normalise for transfection efficiency, the quotient of the luciferase and β-galactosidase activity measurements was calculated for each sample. Data were expressed as mean values ± S.E.M. from three or more experiments. Statistical analyses were done with the SIGMA STAT software (SPSS Science Software GmbH, Erkrath, Germany).

### Nuclear extracts and electrophoretic mobility shift assays (EMSA)

Bovine cotyledons were obtained from a local slaughterhouse. Small peaces of tissue were frozen in liquid nitrogen, and then stored at -80°C until use. Nuclear protein extracts were prepared essentially as described [29]. The protein content of nuclear extracts was determined by the Bradford method. The E-box (-340) probe was prepared by primer extension [30] of the annealed oligonucleotides 5'-AGGGGATTGGGCCACATGACCTCTTTGAGC-3'/5'-GCTCAAAGAGGT-3' using [α^32^P]dCTP and Klenow polymerase (the E-box motif is underlined). Binding reactions contained 35 fmol of labelled DNA, and 2 μg of nuclear extract in a total volume of 10 μl binding buffer (20 mM Tris/HCl pH 7.9, 70 mM KCl, 1 mM EDTA, 5 mM DTT, 0.05% NP-40, 0.01 mg/ml poly d[I-C], 10% glycerol). To demonstrate the specificity of DNA/protein complexes a 100-fold excess of unlabelled double-stranded competitor oligonucleotides was added to the reaction mixtures (E-box; E-box_mut_, 5'-AGGGGATTGGGCAAATTTACCTCTTTGAGC-3', the mutated E-box is underlined). During the supershift assays, 4 μg of polyclonal USF1 or USF2 antibodies (sc-8983X and sc-861X, respectively, Santa Cruz) were added to the binding reactions. After incubation at room temperature for 20 min, samples were subjected to electrophoresis through 6% native polyacrylamide gels in 0.5× TBE. Gels were analysed on a STORM 840 Phosphor Imager using the Image Quant software (Molecular Dynamics, Krefeld, Germany).

### Enrichment of the E-BP from bovine placental nuclear extracts

Double-stranded target oligonucleotides were prepared by annealing equal amounts of the respective HPLC-purified sense-, and biotinylated anti-sense strands (E_wt_, 5'-GCAAGTCATGTGGCCCACACACACACACACACACACACAC-3'/5'-biotin-GTGTGTGTGTGTGTGTGTGTGTGTGGGCCACATGACTTGC-3'; E_mut_, 5'-GCAAGTAAATTTGCCCACACACACACACACACACACACAC-3'/5'-biotin-GTGTGTGTGTGTGTGTGTGTGTGTGGGCAAATTTACTTGC-3'; the E-box and the mutated E-box are underlined). Target oligonucleotides were then immobilised on Dynabeads Streptavidin (Dynal, Hamburg, Germany) according to the supplier's protocol, using 40 pmol of target DNA/1 mg of Dynabeads Streptavidin. Nuclear extracts were prepared as described [29] and heat-treated by incubation at 65°C for 10 min in a heating block. Extracts were then subjected to centrifugation at 14,000 rpm for 10 min in an Eppendorf 5402 centrifuge set to 4°C. Supernatants were dialysed against dialysis buffer (20 mM Tris/HCl pH 7.9, 70 mM KCl, 1 mM EDTA, 10% glycerol) in a Plus One mini dialysis device (GE Healthcare, Freiburg, Germany). The first step in developing the procedure for enrichment of the E-BP was to determine the amount of nuclear extract required to saturate the immobilised Ewt-targets. To this end a series of small scale binding reactions was set up with increasing amounts of heat-treated proteins and a constant concentration of Ewt-targets. After incubation for 20 min at room temperature, protein-bound targets were pelleted and each of the supernatants was then tested by EMSA. The amount of added protein was defined to be saturating when a specific binding complex became visible. Preparative experiments were scaled up accordingly and contained 1 mg of Ewt-Dynabeads in a total volume of 500 μl binding buffer. The protein-bound Dynabeads were washed twice with 1 ml binding buffer. Finally, bound proteins were eluted with 50 μl elution buffer (1 M NaCl, 20 mM Tris/HCl pH 7.9, 1 mM EDTA, 5 mM DTT, 0.05% NP-40, 10% glycerol, 0.5 mg/ml BSA), and dialysed against dialysis buffer. Parallel experiments with the immobilised Emut-targets were performed as a control.

### SDS-PAGE and Western blotting

Proteins were resolved in conventional 12.5% Laemmli gels (acrylamide/bisacrylamide = 30/0.8) at 30 mA per gel, using the Mighty Small SE 250 system (Hoefer, Amersham Biosciences, Freiburg, Germany). Separated proteins were blotted onto PVDF membranes (Millipore, Schwalbach, Germany) using a semi dry blotting apparatus with a three buffer system (anode buffer 1: 0.3 M Tris/HCl pH 10.4, 10% methanol; anode buffer 2: 0.25 mM Tris/HCl pH 10.4, 10% methanol; cathode buffer: 25 mM Tris/HCl pH 9.4, 40 mM 6-amino-n-caproic acid, 10% methanol) for one hour at 1 mA per square centimetre membrane. To prevent non-specific binding, the free binding sites of the membranes were saturated with 5% fat free dry milk powder in TTBS (1 hour at room temperature). Thereafter, membranes were thoroughly washed tree times, each for 10 min with 50 ml TTBS. After each incubation, the membranes were washed in the same way. The antibody dilutions for USF1 and USF2 were 1:2000. They were incubated overnight at 4°C with gentle agitation. This was followed by incubation of the blots with the secondary antibody (goat anti-rabbit HRP labelled, diluted 1:5000, incubation 1.5 h at room temperature). The membranes were washed again and the segregated proteins were subsequently visualised with a chemiluminescence kit (ECL) (Amersham Pharmacia Biotech, Freiburg, Germany) on x- ray film.

### RNA preparation, cDNA synthesis and quantitative real-time PCR (qPCR)

Total RNA from Jeg3 human trophoblast cells (ECACC No. 92120308) was isolated with the RNeasy mini kit (Qiagen, Hilden, Germany). Following primers were used for USF cDNA synthesis and PCR: hsUSF1_RT_, 5'-AAGTGGGGCAGTGAAGGAAAG-3'; hsUSF1_for_, 5'-GCACTCAGGCCTGTGAATCAGGAGA-3'; hsUSF1_rev_, 5'-ATGCTGGCAATAGCCACACTGGTTG-3'; hsUSF2_RT_, 5'-CATGTGTCCCTCTCTGTGCTAAG-3'; hsUSF2_for_, 5'-GATCGTCCAGCTTTCGAAAATCATTC-3'; hsUSF2_rev_, 5'-TCATTCTTCAGCTCCTCGATCTG-3'. For cDNA synthesis, total RNA was reverse transcribed using M-MLV reverse transcriptase, RNase H Minus, Point Mutant (Promega) and cDNAs were cleaned with the High Pure PCR Product Purification Kit (Roche, Mannheim, Germany). qPCR was performed with the LC 480 SYBR Green I Master Kit (Roche) in a LightCycler 480 instrument (Roche) under the following cycling conditions: Pre-incubation at 95°C, 5 min, denaturation at 95°C, 20 s, annealing at 60°C, 15 s, extension at 72°C, 15 s, for 40 cycles. Melting curves were analysed to investigate the specificity of PCR reactions. Cloned amplimeres of the USF genes were used to generate external standard curves. Routinely, standards covering five orders of magnitude (5 × 10^-16 ^to 5 × 10^-12 ^g DNA/reaction) were co-amplified during each run. Copy numbers were calculated relative to the amount of total RNA previously subjected to cDNA synthesis.

### RNAi-mediated knockdown of USF1 and USF2

Jeg3 cells were grown in MEM (Eagle's medium with Earle's salts and non-essential amino acids) supplemented with 1 mM sodium pyruvate and 10% foetal calf serum (Biochrom, Berlin, Germany). The siGenome ON-Target plus SMART pools targeting human USF1 (L-003617) and USF2 (L-003618), and the ON-Target plus siControl non-targeting pool were obtained from Dharmacon (Bonn, Germany). For USF knockdown experiments 1 × 10^4 ^Jeg3 cells/well were seeded in 24-well plates, in serum- and antibiotic free medium, and transfected with a total of 100 nM siRNA along with 250 ng of the P1.1 reporter plasmid, using 0.5 μl Dharmafect Duo transfection reagent. Cells were harvested 72 h after transfection and analysed for luciferase activity.

## Authors' contributions

RF conceived the experimental design of the study, carried out the protein purification and EMSA experiments, interpreted the data and drafted the manuscript. WT participated in the purification of USF proteins, carried out the Western blot analyses and helped to draft the manuscript. JV participated in the design of the study and in the interpretation of the data, and helped to draft the manuscript. All authors read and approved the final manuscript.
